# COVID-19-induced Acute Psychosis Resulting in a Suicide Attempt: A Case Report

**DOI:** 10.5811/cpcem.1401

**Published:** 2023-05-26

**Authors:** Alexander Piszker, Nicholas McManus

**Affiliations:** Michigan State University College of Osteopathic Medicine, Trinity Health Muskegon Emergency Medicine Residency, Muskegon, Michigan

**Keywords:** Suicide attempt, COVID-19, psychosis, emergency, stabbing, case report

## Abstract

**Introduction:**

Psychosis associated with coronavirus disease 2019 (COVID-19) has been previously, but infrequently, reported in the literature. We present a rare case of COVID-19-associated severe psychosis and suicide attempt in an 80-year-old male with no personal or known family history of psychiatric disease. Our patient’s symptoms appeared to be longer lasting than most other reported cases in the available literature.

**Case Report:**

After a COVID-19 diagnosis, our patient experienced fluctuating, long-lasting psychiatric symptoms over a six-month period. During this time, he was unable to function independently. Suggested mechanisms involve a multifactorial combination of neuroinflammation and increased societal stress due to the direct and indirect effects of the virus, respectively.

**Conclusion:**

More research is needed to help identify risk factors, prognostic indicators, and a standard of care for psychosis associated with COVID-19.

## INTRODUCTION

Since the beginning of the global coronavirus disease 2019 (COVID-19) pandemic, adults aged ≥65 years old have accounted for a disproportionate amount of COVID-19- related morbidity and mortiality.^1^ The severe acute respiratory syndrome coronavirus (SARS-CoV) epidemic in 2003 saw case associations with psychiatric manifestations;^2^ likewise, the COVID-19 pandemic shares similar manifestations. There is an increased risk (5.8% above baseline) of new psychiatric diagnoses in the 90 days after a COVID-19 infection.^3^ The risk of new psychiatric symptoms is greater for those patients with a family history of psychiatric conditions.^3^ We present the rare case of COVID-19-associated severe, prolonged psychosis with suicide attempt in an 80-year-old male with no known personal or family history of psychiatric disease.

## CASE REPORT

The patient was an 80-year-old male with relevant past medical history including hypertension, hyperlipidemia, chronic kidney disease stage IIIa, and he was without documented psychiatric history. He initially presented to the emergency department (ED) via ambulance for three days of generalized weakness. He was given one liter of normal saline en route by paramedics. At that time, the patient’s electrocardiogram showed sinus bradycardia, chest radiograph (CXR) was unremarkable, and the comprehensive metabolic panel (CMP), complete blood count (CBC), troponin, B-type natriuretic peptide, and thyroid studies were all unremarkable as well. Aside from sinus bradycardia, his other vital signs were within normal limits. He did test positive for COVID-19, and he qualified for monoclonal antibody therapy. At that time, he was determined to be stable for discharge home.

Three days later (COVID day 6), he presented to a different ED, again for a chief complaint of generalized weakness. He again received intravenous (IV) fluids, was found to be vitally stable, and had an unremarkable laboratory work-up similar to his previous ED visit. He was discharged home with a prescription for dexamethasone (which he did not fill). Three days later (COVID day 9), he presented again to the ED for a chief complaint of generalized weakness and altered mental status. Regarding his altered mental status, he reported intermittent confusion and hallucinations involving seeing wolves coming after him. His vital signs all were within normal limits.

The patient received a broad laboratory and imaging workup including CBC, CMP, troponin, lactate, C-reactive protein, thyroid studies, and blood cultures, all of which were unremarkable. Additionally, a lumbar puncture was performed with cell count, glucose, protein, and Gram stain all returning unremarkable. A computed tomography (CT) of the head and CXR were also performed and resulted without emergent findings. The patient was admitted to the hospital at this visit and observed overnight. He was observed to have waxing and waning mental status. At his best he was fully alert and oriented, while at worst he did have intermittent delusions and hallucinations involving wolves. He did maintain insight into his hallucinations and realized they were not real.

The patient’s daughter flew in from out-of-state and agreed to stay with her father after discharge for a few weeks to ensure primary care and neuropsychology follow-up. The patient was discharged on COVID day 10. Later that same day, he presented to the ED as a priority one trauma activation. The patient had multiple self-inflicted stab wounds to the abdomen and one to the anterior neck. His daughter had called emergency medical services after finding him in the basement with an eight-inch steak knife protruding from his abdomen. The patient had removed the knife from his abdomen and attempted to stab himself in the neck before his daughter was able to take control of the knife. The patient was unable to provide much history on arrival; however, he stated he had done this because he was “a horrible person.”

Initial vital signs showed a blood pressure of 211/116 millimeters of mercury (mm Hg), a temperature of 36.2° C, bradycardia at 53 beats per minute, a respiratory rate of 22 breaths per minute, and an oxygen saturation of 97% on room air. The patient had a stab wound overlying the anterior neck; however, his airway was intact and there was no pulsatile hematoma, no subcutaneous crepitus, or stridor, although his voice was hoarse. There were multiple stab wounds across the abdomen with an expanding hematoma over the right lower quadrant. There was no active external hemorrhage, and the patient was without peritoneal signs. Initial interventions included a tetanus booster, 1 liter of normal saline, 2 grams of ceftriaxone, and 500 milligrams of metronidazole, all administered intravenously. The patient was hemodynamically stable for CT. A CT angiogram of the neck was performed and showed no extravasation of contrast through the great vessels or common carotid arteries. Computed tomography of the chest with IV contrast showed free air consistent with penetrating injury (Image).

An emergent exploratory laparotomy was performed, as well as closure of the abdominal wounds. Eleven stab wounds to the abdomen were noted; six of them were full thickness entering the peritoneal cavity. A thorough exploration of the abdomen revealed no injury to the liver, stomach, small bowel, or colon. The rectus sheath hematomas were decompressed, and the wounds closed.

*CPC-EM Capsule*

What do we already know about this clinical entity?

*Coronavirus disease of 2019 (COVID-19) has case associations with psychiatric manifestations. The risk of new psychiatric diagnoses within 90 days of a COVID-19 infection is 5.8% above baseline*.

What makes this presentation of disease reportable?

*Our patient, without history of previous psychological disease, lost six months of functional status secondary to his COVID- 19-related psychosis*.

What is the major learning point?

*As seen with our patient, the COVID-19- associated psychiatric symptoms can be severe enough to progress to a suicide attempt and their course can be prolonged*.

How might this improve emergency medicine practice?

*Recognizing that agitation and delirium are associated with COVID-19 infections may help guide testing and treatment in patients with similar clinical pictures*.

After stabilization by the trauma team, he was evaluated by psychiatry. At that time (COVID day 11), he continued to endorse suicidal ideation, visual hallucinations, and depressive symptoms. Inpatient psychiatric placement was recommended. He was started on sertraline. The patient continued to express suicidal ideation throughout his hospital stay with documented quotes such as “I am nothing but evil and do not deserve to be living.” On COVID day 15 he was discharged from the hospital to inpatient psychiatric care.

On COVID day 18, while at the inpatient psychiatric facility, he was found to have an acute kidney injury and was transferred back to the hospital for medical management. There he was evaluated by neuropsychology and had an unremarkable magnetic resonance image (MRI) of the brain. Over the next 14 days, he had escalating anxiety that was unresponsive to quetiapine and haloperidol. Benzodiazepine administration worsened the patient’s delirium. The patient’s quetiapine was discontinued, and his regimen was changed to olanzapine and hydroxyzine.

On COVID day 29 the patient had an abrupt, significant improvement in mentation where he was calm, cooperative, and appropriately conversive. Over the next seven days, he continued to have waxing and waning mental status. Sodium valproate was briefly initiated; however, he developed a papular, erythematous rash shortly after, and this was discontinued. Mirtazapine was then added to the patient’s regimen, and his sertraline dose was increased. By COVID day 43 the patient had significant improvements in his mental status and depressive symptoms. He took a mini-mental state examination and scored 24 of 25, which is within the normal range. He also took a St. Louis University Mental Status Examination and scored 20 of 30, which is consistent with cognitive impairment and dementia. On COVID day 44 he was discharged to adult foster care with 24-hour supervision on a medication regimen of olanzapine, mirtazapine, and sertraline.

He was moved into an adult foster-care home with close outpatient follow-up with his family medicine physician. After multiple follow-up visits, interval hospitalization, and serial medication adjustments, the patient’s mental status stabilized, and he was discharged from adult foster care. He is maintained on sertraline, olanzapine, and mirtazapine. He now lives safely on his own, plays golf multiple times a week, and can care for his pet dog. Despite the eventual, significant resolution in our patient’s symptoms, he ultimately lost six months of functional status secondary to his COVID-19-associated psychosis.

## DISCUSSION

Although this case is similar to existing cases in the literature, the patient demographics and presentation are unique. This case adds to existing literature surrounding the elderly population and COVID-19. The exact mechanism of this patient’s psychosis is unknown. There was no documented personal psychiatric history, history of suicidal ideation, or reported significant family psychiatric history. Furthermore, our patient did not fill his dexamethasone prescription; so the likelihood of medication-induced psychosis is very low. Our patient’s CT of the brain, MRI of the brain, and cerebral sinus fluid studies did not show any evidence of neuroinflammation or neurologic injury; however, an extended encephalitis panel was not performed.

A review of the available literature revealed common psychiatric symptoms associated with COVID-19 such as anxiety and depression, as well as more severe manifestations such as delusions, paranoia, and suicidal thoughts.^4^ Similar to what we report regarding our patient, other case reports have documented new-onset psychiatric symptoms in patients without a previous psychiatric history.^4^–^7^ While some of the reported cases of new-onset psychiatric symptoms did not progress to a suicide attempt, there are reports of progressive psychosis leading to suicide attempts similar to our patient. Special attention is paid to two cases involving attempted suicide by neck laceration, similar to our patient although these patients were much younger (37 and 52 years old, respectively).^8^,^9^

SARS-CoV-2 is known to cause large-scale inflammatory responses, sometimes referred to as “cytokine storm,” which studies have implicated in the pathogenesis of psychiatric symptoms in acute COVID-19.^10^,^11^ Other authors suggest a more multifactorial mechanism for the pathogenesis of new-onset psychiatric symptoms associated with a COVID-19 infection. These authors take into consideration both the neuroinflammation and the psychosocial effects of physical/social isolation, hospitalization and its associated procedures, and the sociocultural effects of the pandemic.^12^ Specifically regarding our patient, a possible link to the development of new-onset psychiatric symptoms in the peri-COVID-19 setting could be his chronic kidney disease. Researchers in the United Kingdom suggest a link between kidney disease patients and mental health problems, suggesting a risk increase of 100% compared to the general population.^13^ Otherwise, there were no other social stressors (eg, no known financial difficulties, loss of a loved one, or illicit substance use) apparent in our patient’s history.

While there certainly are multiple reports of new-onset psychiatric symptoms in patients without previous psychiatric history,^4^–^7^ there are also reports highlighting the onset of COVID-19-associated psychiatric symptoms in patients with either personal or family history of psychiatric symptoms.^7^,^14^ Additionally, our patient had a protracted course of his psychosis and recurrent agitation and delirium. This is incongruent with most cases reported in the literature. In one retrospective study of patients with new-onset psychotic symptoms and confirmed diagnosis of SARS-CoV-2, excluding patients with a previous personal or family history of severe mental disorders, 80% of the patients experienced resolution of their symptoms in less than two weeks.^15^ Even though the overall number of patients studied was limited, data suggests that in a majority of patients their psychiatric symptoms are not long-lasting. However, due to the recent onset of the phenomena described in this report, the optimal treatment regimen is still unknown and can include a variety of medications.

## CONCLUSION

Additional research is needed to delve into this complication of COVID-19, as reported cases in the literature are still quite rare. It remains unknown whether psychosis associated with COVID-19 requires a different standard of care compared to other causes of delirium and psychosis.

## Figures and Tables

**Image:**
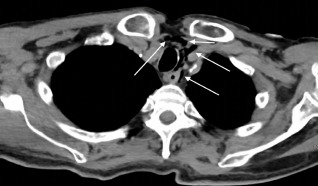
Computed tomography of the chest with contrast showing free air (arrows) adjacent to the trachea contained within the mediastinum secondary to the self-inflicted stab wound to the patient’s neck.
